# Factors Associated with Self-Harm Attempts Among Female Adolescents with Self-Reported Reclusive Tendencies in Republic of Korea

**DOI:** 10.3390/children13091224

**Published:** 2026-09-10

**Authors:** So-Hyun Moon, Hyeon Na, A Ra Kim

**Affiliations:** 1 Faculty of Nursing, Chosun University, Gwangju 61452, Republic of Korea; 2 Nurse of Pediatrics, Mokpo Miz-I Hospital, Mokpo 58684, Republic of Korea; 3 Faculty of Nursing, Seoyeong University, Gwangju 61268, Republic of Korea; mniara@seoyeong.ac.kr

**Keywords:** female, adolescent, self-reported reclusive tendencies, self-harm attempts, smartphone

## Abstract

**Highlights:**

**What are the main findings?**

- Among female adolescents with self-reported reclusive tendencies who utilize crisis support services, smartphone overdependence demonstrated an inverse multivariable association with self-harm attempts. However, this preliminary finding should be interpreted strictly as an exploratory hypothesis rather than evidence of a protective effect.
- Personal psychological factors (depression, self-esteem, self-control) and parent–child relationships are strongly associated with self-harm attempts in this vulnerable population.

**What are the implications of the main findings?**

- Future research must rigorously investigate the exact role of digital engagement among isolated youth using validated clinical tools, recognizing that our inverse association is strictly an exploratory hypothesis likely influenced by statistical suppression and unmeasured confounding rather than a genuine protective effect.
- Prevention and intervention strategies for reclusive female youth should prioritize managing depression, enhancing self-control, strengthening self-esteem, and supporting parent–child dynamics.

**Abstract:**

Background/Objectives: Self-harm attempts among adolescents represent a major public health concern, often associated with depression, low self-esteem, and social withdrawal. While excessive smartphone use is generally linked to internalizing distress, digital engagement might play complex roles among socially isolated youth. This study examined factors associated with self-harm attempts among female adolescents with self-reported reclusive tendencies in South Korea, with a specific focus on evaluating the exploratory role of smartphone overdependence. Methods: Secondary data analysis was conducted on 893 female adolescents with self-reported reclusive tendencies who utilized or were admitted to crisis youth support organizations, drawn from the nationwide Survey on the Living Conditions of Users of Crisis Youth Support Organizations. Statistical procedures incorporated complex-sample descriptive statistics, Rao–Scott chi-square tests, and complex-sample hierarchical logistic regression. Results: In univariate analyses, economic status, self-esteem, self-control, depression, and parent–child relationships differed significantly according to self-harm history. In multivariable hierarchical logistic regression, higher self-esteem (OR = 0.44, *p* < 0.001) and positive parent–child relationships (OR = 0.57, *p* = 0.004) were associated with lower odds of self-harm attempts, whereas depression (OR = 2.23, *p* = 0.001) and lower self-control (OR = 1.40, *p* = 0.009) were associated with higher odds. The inclusion of smartphone overdependence in Step 3 (yielding a modest increase in explanatory power, ΔR^2^ = 0.02) revealed that being in the smartphone overdependence risk group was associated with lower odds of self-harm attempts (OR = 0.55, *p* = 0.003). Conclusions: Self-harm attempts in this service-using population are strongly associated with core psychological and familial distress. The inverse multivariable association regarding smartphone overdependence is preliminary and hypothesis-generating; it may reflect statistical suppression, unmeasured confounding, or indirect digital connection among isolated youth, rather than a direct protective function. Findings apply specifically to crisis-service-using female youth and underscore the need for longitudinal research with refined clinical measures.

## 1. Introduction

Adolescents with self-reported reclusive tendencies describe a condition in which individuals tend to avoid social relationships and minimize contact with the outside world [1]. Originally recognized as a prominent social concern in East Asia, social withdrawal among youth is now recognized as an international public health challenge [1,2,3].

According to the 2022 Crisis Youth Support Organization User Survey in South Korea, 46.7% of respondents reported staying home for extended periods without outdoor activity in the past year, with female respondents showing a higher prevalence of reclusive tendencies than males (49.4% vs. 44.2%) [4]. Furthermore, approximately 140,000 youth aged 13–18 are estimated to be reclusive [5].

Although psychiatric communities in South Korea have examined reclusive youth since the early 2000s, early approaches largely focused on individual pathology and psychological problems [6]. However, these individuals face profound challenges—such as instability, social maladjustment, family breakdown, and educational disruption—frequently resulting in severe depression [7]. Consequently, recognizing the critical need for active national intervention, reclusive youth have increasingly been incorporated into specialized welfare initiatives and designated as special support targets during the 2023 national crisis [8]. In this secondary analysis, we focused a priori on female adolescents because national crisis survey data indicate higher rates of reclusive tendencies and self-harm attempts among adolescent girls relative to boys [4,9,10]. Adolescent girls exhibit heightened vulnerability to internalizing distress, depression, and mood dysregulation [9,10]. Understanding factors associated with self-harm attempts in this specific population is vital for designing effective community support systems.

Reclusive youth frequently experience mental health problems, such as social withdrawal, anxiety, and helplessness [11]. Female adolescents with reclusive tendencies often lack social skills and struggle to build social relationships, tending to withdraw when faced with excessive stress [12]. They also exhibit impulsive and defensive traits during self-harm attempts [13]. Notably, the overall prevalence of self-harm attempts is 30.7% higher among female adolescents than males (49.0% vs. 19.1%), and past-year prevalence is approximately 3.6 times higher in females (29.8% vs. 8.2%) [14].

Previous studies have shown that adolescent self-harm is closely linked to diminished self-esteem, impaired self-control, and unsupportive parent–child relationships [15,16,17,18,19,20,21,22]. Among adolescents, 40.1% are at risk of smartphone dependence compared with 22.7% among adults [23]. In particular, girls were more likely to own a cell phone (94.8%) than boys (88.3%), and 18.7% were severely dependent [24]. In parallel, excessive smartphone use is widely recognized as a risk factor for internalizing problems [25,26,27]. However, for socially withdrawn youth who face severe real-world social barriers, digital environments might theoretically present alternative avenues for low-pressure communication or information access.

Importantly, our investigation into smartphone overdependence was conceptualized as a non-directional, exploratory analysis. Given the limited literature on reclusive youth, we aimed to examine whether digital engagement is associated with self-harm attempts in this crisis-service-using cohort, without assuming a direct protective or harmful mechanism. Ultimately, this study aims to identify personal, familial, and behavioral factors associated with self-harm attempts among female adolescents with self-reported reclusive tendencies using crisis support services.

## 2. Methods and Materials

### 2.1. Study Design

This study used a cross-sectional, correlational secondary data analysis design, examining raw data from the ‘Survey of Living Conditions of Users of Crisis Youth Support Organizations’ conducted in South Korea.

### 2.2. Participants

Data for this study were obtained from the ’Survey on the Living Conditions of Users of Crisis Youth Support Organizations,’ conducted by the Korean Ministry of Gender Equality and Family in accordance with raw data disclosure regulations. Conducted between January and December 2021, the survey utilized a stratified cluster sampling method to establish a target population of youths who utilized or were admitted to crisis youth support organizations. With participant safety prioritized, surveys were administered by institutional research staff through direct visits and self-administered questionnaires or postal collection. Of the 4399 initial respondents, data from 4203 youths—including 893 female adolescents with self-reported reclusive tendencies—were included in the final analysis. This study was approved by the ethical review board of University C (IRB No. 2-1041055-AB-N-01-2023-46).

### 2.3. Variables

The variables analyzed in this study were derived from the structured questionnaires of the survey (Supplementary Materials).

### 2.4. General Characteristics

Sociodemographic covariates included age category (12–15 years vs. 16–18 years), school enrollment status, dropout history, career plans, health checkup status, nationality, father’s/mother’s nationality, and household economic level (5-point scale from ‘Very poor’ to ‘Very well off’).

### 2.5. Reclusive Tendencies

Reclusive tendencies were operationalized using a single dichotomous screening item from the national survey: ‘In the past year, have there been times when you stayed home without going outside or engaging in outside activities?’ (1 = Yes, 2 = No). This item served as a non-clinical proxy for homebound status.

### 2.6. Experience of Self-Harm Attempts

Self-harm attempts were assessed using a survey item asking whether participants had attempted self-harm in the past year. Responses were originally collected on a frequency scale (1 = 1–2 times per week, 2 = 1–2 times per month, 3 = 1–2 times per year, 4 = none in the past year but previously, and 5 = never in a lifetime) and subsequently recoded into a binary variable (1–4 = Yes, 5 = No) for analysis.

### 2.7. Self-Esteem

Self-esteem was measured using 4 items rated on a 4-point Likert scale (1 = Not at all to 4 = Very much so; theoretical sum score range: 4 to 16). For regression modeling, scale sum scores were computed (higher scores indicate higher self-esteem). Cronbach’s α was 0.90.

### 2.8. Self-Control

Self-control was assessed using 5 items rated on a 4-point scale (1 = Not at all true to 4 = Very true; theoretical sum score range: 5 to 20). Sum scores were calculated for multivariable models, with higher scores indicating lower self-control. Cronbach’s α was 0.77.

### 2.9. Depression

During the past year, have you felt so sad or hopeless for two consecutive weeks that it interfered with daily activities? (1 = Yes, 2 = No).

### 2.10. Parent–Child Relationships

Parent–child relationship quality was measured using 3 items on a 4-point Likert scale (theoretical sum score range: 3 to 12; higher sum scores reflect more positive relationships). Cronbach’s α was 0.89.

### 2.11. Parental Neglect Experience

Parental neglect was assessed using 3 items on a 4-point Likert scale (theoretical sum score range: 3 to 12; higher scores indicate higher neglect). Cronbach’s α was 0.55.

### 2.12. Smartphone Overdependence

Smartphone overdependence was measured using the standardized Korean Smartphone Overdependence Scale (10 items, 4-point Likert scale, theoretical sum score range: 10 to 40) [28]. This scale evaluates self-control failure, salience, and daily life conflict associated with phone usage, but does not capture the qualitative content, purpose, or social context of digital use. According to standardized cutoffs, participants were categorized into the ‘Over dependence risk group’ (sum score ≥ 23, combining potential and high risk) or the ‘General group’ (sum score < 23). Cronbach’s α was 0.89.

### 2.13. Data Analysis

Statistical analyses were executed using SPSS version 27.0 with complex-sample analysis modules to incorporate sample weights, stratification, and primary sampling units.

1. Univariate differences in general characteristics and study variables by self-harm history were evaluated using complex-sample Rao–Scott chi-square (χ^2^) tests for categorical variables and *t*-tests for continuous variables.
2. Hierarchical complex-sample logistic regression analyses were performed in three steps: Step 1 (General characteristics), Step 2 (Psychological and familial variables), and Step 3 (Smartphone overdependence risk group).

## 3. Results

### 3.1. Differences in General Characteristics Between Participants with and Without a History of Self-Harm Attempts

Among 893 female adolescents with self-reported reclusive tendencies, 478 (53.5%) reported a history of self-harm attempts. Regarding economic status, self-harm attempts were reported by 34 (75.7%) participants who were very poor, 131 (67.2%) poor, 257 (49.0%) moderate, and 45 (43.8%) well-off, compared with only three (37.5%) very well-off participants, showing a statistically significant difference (χ^2^ = 32.95, *p* = 0.003) (Table 1).

### 3.2. Differences in Variables by Experience with Self-Harm Attempts

Youth with self-harm attempt history exhibited significantly lower self-esteem (t = 11.84, *p* < 0.001) and lower parent–child relationship scores (t = 6.63, *p* < 0.001). Lower self-control (t = −3.14, *p* = 0.005) and presence of depression (F = 42.97, *p* < 0.001) were significantly more frequent in the self-harm attempt group (Table 2).

### 3.3. Factors Associated with Self-Harm Attempts

In Model 1 (General characteristics), sociodemographic factors explained a small proportion of variance (Nagelkerke R^2^ = 0.07). In Model 2 (Psychological and familial variables), explanatory power increased substantially (Nagelkerke R^2^ = 0.25). Higher self-esteem (OR = 0.47, *p* < 0.001) and positive parent–child relationships (OR = 0.57, *p* = 0.005) were associated with significantly lower odds of self-harm attempts, whereas depression (OR = 2.00, *p* = 0.002) was associated with higher odds. In Model 3, the addition of smartphone overdependence showed a minor incremental increase in explanatory power (R^2^ = 0.02, total Nagelkerke R^2^ = 0.27). Higher self-esteem (OR = 0.44, 95% CI: 0.32–0.60, *p* < 0.001) and positive parent–child relationships (OR = 0.57, 95% CI: 0.40–0.82, *p* = 0.004) were significantly associated with decreased odds of self-harm attempts. Depression (OR = 2.23, 95% CI: 1.49–3.41, *p* = 0.001) and lower self-control (OR = 1.40, 95% CI: 1.09–1.78, *p* = 0.009) were significantly associated with increased odds. The smartphone overdependence risk group demonstrated lower odds of self-harm attempts compared to the General group (OR = 0.55, 95% CI: 0.38–0.80, *p* = 0.003) (Table 3).

## 4. Discussion

This study examined personal, familial, and digital factors associated with self-harm attempts in a nationwide sample of female adolescents with self-reported reclusive tendencies utilizing crisis support services. A comparison of the general characteristics of female adolescents with reclusive tendencies who attempted self-harm revealed significant differences in their economic status, with lower economic status being associated with higher rates of self-harm attempts. A comparison of economic status among groups revealed that a higher percentage of respondents who reported being ‘very poor’ and ‘poor’ had a history of self-harm attempts. Park’s study revealed that a lower family economic status was associated with an increase in self-harming behaviors [29], which is consistent with the results of this study.

Hierarchical logistic regression identified self-esteem, self-control, depression, parent–child relationships, and smartphone overdependence as significant factors associated with self-harm attempts. First, low self-esteem was associated with increased odds of self-harm attempts, consistent with prior findings [30]. Adolescence is a critical period for developing self-identity and social relationships, but interpersonal trauma and academic stress can drive youth into reclusion and severely diminish their self-esteem [31,32]. Despite this vulnerability, research shows that reclusive youth possess the inherent resilience to recover their self-esteem through autonomous efforts to overcome their isolation [33,34]. Therefore, rather than merely targeting physical reclusiveness, interventions must empower these individuals to solve intrinsic problems and independently rebuild their confidence.

Second, low self-control was significantly associated with higher odds of self-harm, as adolescents often resort to self-injurious behaviors to exert bodily control when overwhelmed by emotional distress and anxiety [35,36,37]. Conversely, enhancing self-regulation enables individuals to rationally govern their actions, which significantly reduces these maladaptive tendencies [35]. Thus, targeted interventions enhancing self-regulation are essential to prevent self-harm among reclusive female adolescents.

Depression significantly elevates the risk of self-harm, with reclusive adolescents reporting notably higher levels of depressive symptoms compared to the general population [4,38,39]. This psychological distress is particularly critical because adolescent depression frequently becomes chronic, persisting into adulthood and further exacerbating self-injurious behaviors [40]. The underlying causes of depression in this specific demographic are highly complex and difficult to assess due to their severe avoidance of interpersonal relationships. Consequently, comprehensive qualitative research is required to elucidate these unique depressive triggers and develop effective strategies to reduce self-harm attempts.

Fourth, a negative parent–child relationship was significantly associated with a higher likelihood of self-harm. Parental overcontrol and persistent family conflicts often trigger adolescent withdrawal, severely disrupting their developmental need for both autonomy and familial protection [41]. Because parents are frequently the only remaining social contacts for reclusive youth, deteriorating family dynamics can lead to complete social disconnection. Consequently, integrating family counseling and parental support is a critical component of any effective intervention [13].

Finally, the analysis revealed a paradoxical inverse association between smartphone overdependence and self-harm attempts, deviating from the established literature that links problematic digital use to increased self-injurious behaviors [42]. This discrepancy must be interpreted within the specific context of severe physical isolation; for reclusive youth facing profound interpersonal difficulties, smartphones often serve as their primary, or even sole, medium for daily social interaction [43,44]. In such extreme environments, online platforms can provide these highly vulnerable adolescents with a rare, low-barrier avenue for emotional self-disclosure, peer support, and exposure to positive recovery norms [45,46]. Importantly, the scale utilized to measure smartphone overdependence inherently focuses on problematic usage patterns—such as loss of control and interpersonal conflict—rather than the qualitative dimensions of digital engagement, including its specific content or purpose. Therefore, the current data preclude any direct confirmation of whether these adolescents actively utilized their devices for adaptive functions, such as social support, help-seeking, or emotional regulation. Nevertheless, while problematic overdependence remains a critical concern, appropriately designed digital interventions and mobile health applications could potentially be leveraged in the future to safely mitigate emotional isolation and offer accessible coping strategies for this hard-to-reach population [47,48].

Crucially, the inverse association regarding smartphone overdependence should not be interpreted as evidence of a direct protective effect. This interpretation requires considerable caution due to the small increase in explained variance (ΔR^2^ = 0.02). The finding that the effect of smartphone overdependence emerges only in the multivariable model and is absent in univariate comparisons indicates that the association may be statistically fragile and could reflect a suppression effect, a confounding effect, or unmeasured third-variable effects. Therefore, this association remains preliminary, context-dependent, and strictly hypothesis-generating.

Several substantial limitations must be explicitly acknowledged. First, the cross-sectional design precludes causal inferences. Second, reliance on single, non-clinical proxy items for reclusive tendencies and self-harm presents a risk of misclassification bias. Specifically, self-harm was assessed using a single item that conflates lifetime and recent occurrences while lacking critical clinical details (e.g., frequency, method, severity, intent) and standard diagnostic differentiation between non-suicidal self-injury (NSSI) and suicide attempts via instruments like the PHQ-9 or CDI. Furthermore, depression was measured through a coarse single dichotomous proxy for sadness, and ‘reclusive tendencies’ relied on a single non-clinical item that may capture temporary homebound states rather than sustained clinical withdrawal. Third, the neglect scale’s low internal consistency (Cronbach’s α = 0.55) limits statistical power, meaning the irrelevance of parental neglect cannot be prematurely concluded. Fourth, findings are limited to South Korean crisis-service-using female adolescents and cannot be generalized to the general population. Finally, unmeasured confounding factors may have influenced the observed relationship. For instance, severe psychopathology, such as profound depression or anhedonia, could lead to a general withdrawal from all activities—including digital engagement—while independently increasing the risk of self-harm. Additionally, the lack of data regarding the qualitative purpose of smartphone use (e.g., active peer support-seeking versus passive avoidance) limits our ability to rule out third-variable effects. Future longitudinal studies using validated psychiatric assessments are necessary to confirm these exploratory findings.

## 5. Conclusions

Our analysis revealed that personal factors (self-esteem, self-control, depression), family factors (parent–child relationships), and environmental factors (smartphone overdependence) were significantly associated with self-harm attempts in this specific subpopulation. To reduce self-harm attempts in female adolescents with reclusive tendencies, providing individualized support through systematic interventions that address self-esteem, self-regulation, depression, and parent–child interactions is crucial. Furthermore, the exploratory finding regarding smartphone use should be treated strictly as hypothesis-generating. Future research must rigorously investigate the qualitative nature of this digital engagement using refined clinical measures and longitudinal designs, as the current association may reflect statistical suppression or unmeasured confounding rather than a genuine protective effect.

## Figures and Tables

**Table 1:** Univariate analysis of variables related to experience of self-harm attempts (N = 893).

Variables	Categories	Total Sample (n = 893)	Self-Harm Attempts		Rao–Scott *x*^2^	*p*
			Yes (n = 478)	No (n = 415)		
		n (% *)	n (% *)	n (% *)		
Age (year)	12–15	381 (42.5)	201 (51.9)	180 (48.1)	0.77	0.262
	16–18	512 (57.5)	277 (54.9)	235 (45.1)		
Attending school	Yes	427 (47.8)	230 (54.2)	197 (45.8)	0.00	0.989
	No	454 (52.2)	246 (54.2)	208 (45.8)		
Dropping out of school	I have quit before	440 (51.2)	238 (54.0)	202 (46.0)	0.06	0.834
	I have never quit before	433 (48.8)	234 (54.8)	199 (45.2)		
Career planning	Advancement	394 (50.0)	197 (50.2)	197 (49.8)	3.32	0.188
	Economic activity	191 (24.7)	100 (51.8)	91 (48.2)		
	Other	201 (25.3)	115 (58.0)	86 (42.0)		
Health check	Received	469 (53.7)	242 (52.2)	227 (47.8)	2.30	0.376
	Not received	404 (46.3)	227 (56.0)	177 (44.0)		
Nationality	Korea	861 (97.3)	460 (53.4)	401 (46.6)	2.08	0.142
	Other	22 (2.7)	14 (68.3)	8 (31.7)		
Father’s nationality	Korea	850 (97.6)	457 (54.0)	393 (46.0)	0.28	0.569
	Other	21 (2.4)	10 (48.1)	11 (51.9)		
Mother’s nationality	Korea	802 (91.7)	433 (54.4)	369 (45.6)	1.96	0.217
	Other	71 (8.3)	35 (45.9)	36 (54.1)		
Economic level	Very poor	46 (5.2)	34 (75.7)	12 (24.3)	32.95	0.003
	Poor	198 (22.6)	131 (67.2)	67 (32.8)		
	Average	518 (59.1)	257 (49.0)	261 (51.0)		
	Well	106 (12.1)	45 (43.8)	61 (56.2)		
	Very well	9 (1.0)	3 (37.5)	6 (62.5)		
Smartphone overdependence	General group	475 (65.5)	256 (53.9)	219 (46.1)	0.08	0.759
	Overdependence risk group ^+^	411 (34.5)	218 (52.9)	193 (47.1)		

OR = Odd Ratio. * Weighted percentages calculated via complex-sample analysis. Skipped responses excluded. ^+^ Potential risk group + High risk group.

**Table 2:** Differences in variables by experience with self-harm attempts (N = 893).

Variables *		Total Sample (n = 893)	Self-Harm Attempts		t or Adjusted F	*p*
			Yes (n = 478)	No (n = 415)		
		M ± SE or n (%)	M ± SE or n (%)	M ± SE or n (%)		
Self-Esteem		2.73 ± 0.04	2.47 ± 0.04	2.99 ± 0.05	11.84	<0.001
Self-Control		2.34 ± 0.02	2.40 ± 0.03	2.28 ± 0.03	−3.14	0.005
Depression	Yes	392 (43.9)	268 (68.4)	124 (31.6)	42.97 †	<0.001
	No	484 (54.2)	197 (40.7)	287 (59.3)		
Parent–Child Relationship		2.92 ± 0.06	2.68 ± 0.07	3.17 ± 0.06	6.63	<0.001
Neglect Experience		1.75 ± 0.03	1.79 ± 0.04	1.71 ± 0.04	−1.48	0.152
Smartphone Overdependence		2.09 ± 0.02	2.10 ± 0.03	2.09 ± 0.03	−0.35	0.729

SE = Standard Error. † Rao–Scott Adjusted F. * Item-mean scores (1–4 scale).

**Table 3:** Factors associated with self-harm attempts among female adolescents with self-reported reclusive tendencies (N = 893).

Categories (Reference)		Model 1				Model 2				Model 3			
		OR	95% CI		*p*	OR	95% CI		*p*	OR	95% CI		*p*
			Low	High			Low	High			Low	High	
Constant		-	-	-	0.191	-	-	-	0.001	-	-	-	0.001
Age(year)	12–15	1.01	0.76	1.33	0.971	1.12	0.77	1.62	0.535	1.14	0.80	1.62	0.446
	16–18	Reference											
Attending school	Go	1.22	0.61	2.48	0.559	1.02	0.42	2.49	0.964	1.05	0.44	2.51	0.904
	Doesn’t go	Reference											
Dropping out of school	I have quit before	1.01	0.52	1.94	0.987	1.17	0.52	2.64	0.692	1.14	0.51	2.57	0.737
	I have never quit before	Reference											
Career planning	Advancement	0.77	0.1	1.18	0.217	0.93	0.59	1.45	0.729	0.99	0.66	1.47	0.949
	Economic activity	0.76	0.52	1.11	0.149	0.80	0.44	1.45	0.445	0.89	0.50	1.60	0.691
	Other	Reference											
Health check	Received	0.81	0.53	1.23	0.300	0.90	0.65	1.24	0.507	0.90	0.630	1.28	0.525
	Not received	Reference											
Economic level	Very poor	5.40	0.84	34.56	0.073	1.07	0.19	6.11	0.940	1.12	0.22	5.86	0.885
	Poor	3.85	0.53	28.18	0.174	0.91	0.14	5.73	0.912	0.94	0.16	5.49	0.944
	Average	1.59	0.23	11.14	0.630	0.53	0.09	3.12	0.462	0.53	0.10	2.87	0.441
	Well	1.10	0.17	6.92	0.918	0.46	0.10	2.17	0.313	0.51	0.12	2.14	0.339
	Very well	Reference											
Self-esteem						0.47	0.35	0.64	<0.001	0.44	0.32	0.60	<0.001
Self-control ^†^						1.31	0.97	1.78	0.077	1.40	1.09	1.78	0.009
Depression	Yes					2.00	1.33	3.00	0.002	2.23	1.49	3.41	0.001
	No	Reference											
Parent–child relationship						0.57	0.39	0.82	0.005	0.57	0.40	0.82	0.004
Neglect experience						0.71	0.49	1.03	0.073	0.72	0.49	1.06	0.096
Smartphone overdependence	Overdependence risk group ^‡^									0.55	0.38	0.80	0.003
	General group	Reference											
Cox & Snell R^2^		0.05				0.19				0.20			
Nagelkerke R^2^		0.07				0.25				0.27			

OR = odds ratio; CI = confidence interval. Adjusted Demographic factor; Age, Institutions type, Residential Type. ^†^ higher scores represent lower levels of reported self-control. ^‡^ Potential risk group + High risk group.

## Data Availability

The data are available upon request from the Survey of Living Conditions of Users of Crisis Youth Support Organizations, Ministry of Gender Equality, and Family.

## Supplementary Materials

The following supporting information can be downloaded at: https://www.mdpi.com/article/10.3390/children13091224/s1, Table S1. Measurement Items and Survey Details.

## Abbreviations

The following abbreviations are used in this manuscript:

MOGEF	Ministry of Gender Equality and Family
