# Supplementary material for: Factors Associated with Self-Harm Attempts Among Female Adolescents with Self-Reported Reclusive Tendencies in Republic of Korea
Source: Children (Basel). 2026 Sep 10;13(9):1224. doi: 10.3390/children13091224 (PMC13605861; doi:10.3390/children13091224)
Supplement: Supplementary file 1 [file children-13-01224-s001.zip › children-4318673-supplementary.pdf]

Table S1. Measurement Items and Survey Details

Variable	Original Primary Survey Item / Wording	Response Options / Coding	Score Range
Reclusive Tendencies	In the past year, have there been times when you stayed home without going outside or doing any outside activities?	1 = Yes, 2 = No	Binary proxy
Self-Harm Attempts	Have you ever attempted self-harm in the past year?	1 = 1–2 times per week, 2 = 1–2 times per month, 3 = 1–2 times per year, 4 = None in the past year but previously, 5 = Never in a lifetime	Recoded into a binary variable (Scores 1–4 = Yes, Score 5 = No
Depression	During the past year, have you felt so sad or hopeless for two consecutive weeks that it interfered with daily activities?	1 = Yes, 2 = No	Binary proxy
Self-Esteem	4 items (e.g., 'I am a valuable person', 'I am satisfied with myself')	1 = Not at all to 4 = Very much so	Sum: 4–16 (Mean presented in Table 2)
Self-Control	5 items (e.g., 'I act impulsively', 'I get angry easily')	1 = Not at all true to 4 = Very much so	Sum: 5–20 (Higher = lower control)
Parent–Child Relationship	3 items (e.g., 'My parents make me feel loved and cared for')	1 = Not at all to 4 = Very much so	Sum: 3–12
Smartphone Overdependence †	10 items from standardized Korean Smartphone Overdependence Scale [28]	1 = Not at all to 4 = Very much so	Sum: 10–40<br>Sum score range: 10–40.

†Categorized into the General Group (< 23) and the Over-dependence Risk Group (≥23, combining potential-risk [23–30] and high-risk [≥31] groups
